# Obesity-Related Serum Monocyte Chemoattractant Protein-1 (MCP-1) as a Biomarker of Plaque Instability in Patients Undergoing Carotid Endarterectomy

**DOI:** 10.3390/ijms26104731

**Published:** 2025-05-15

**Authors:** Adam Płoński, Anna Krupa, Adam Filip Płoński, Dariusz Pawlak, Marcin Gabriel, Beata Sieklucka, Jerzy Głowiński, Krystyna Pawlak

**Affiliations:** 1 Department of Vascular Surgery and Transplantation, Medical University of Bialystok, 15-276 Bialystok, Poland; adam.plonski@sd.umb.edu.pl (A.F.P.); jglow@wp.pl (J.G.); 2 Department of Internal Medicine and Metabolic Diseases, Medical University of Bialystok, 15-276 Bialystok, Poland; anna.krupa@umb.edu.pl; 3 Department of Pharmacodynamics, Medical University of Bialystok, 15-222 Bialystok, Poland; dariusz.pawlak@umb.edu.pl; 4 Department of Vascular and Intravascular Surgery, Angiology and Phlebology, Poznań University of Medical Science, 61-701 Poznan, Poland; mgabriel@pro.onet.pl; 5 Department of Monitored Pharmacotherapy, Medical University of Bialystok, 15-222 Bialystok, Poland; beata.sieklucka@umb.edu.pl (B.S.); krystyna.pawlak@umb.edu.pl (K.P.)

**Keywords:** atherosclerosis, carotid plaque, carotid endarterectomy, MCP-1, monocyte chemoattractant protein-1, gray-scale median

## Abstract

Atherosclerosis is a major contributor to ischemic stroke. Carotid plaque instability is a critical determinant of cerebrovascular events, yet its identification remains challenging. One chemokine with well-documented proatherogenic properties is MCP-1, whose levels are elevated in patients with conditions such as hypertension, obesity, and atherosclerosis. This study evaluated the association between obesity, serum MCP-1 levels, and carotid plaque instability as determined by ultrasound gray-scale median (GSM) analysis in 77 patients who underwent carotid endarterectomy. Patients were classified by body mass index. Serum MCP-1 concentrations were measured using the enzyme-linked immunosorbent assay technique. Analyses were performed to explore relationships between clinical parameters, biochemical markers, and plaque stability. Increasing body weight was paralleled by higher MCP-1 levels and lower GSM values, indicative of unstable plaques. Moreover, logistic regression analysis identified MCP-1 as one of the independent predictors of plaque instability, particularly in overweight and hypertensive patients. These results indicate the potential usefulness of MCP-1 as a biomarker of carotid plaque instability, confirming the negative effect of obesity in promoting other known cardiovascular risk factors causing plaque instability in patients with carotid atherosclerosis.

## 1. Introduction

Carotid atherosclerosis is a degenerative-inflammatory process that leads to plaque formation, causing arterial narrowing and cerebral ischemia. Importantly, the rupture of unstable plaques—characterized by a lipid-rich, soft necrotic core and inflammatory cells—can trigger an ischemic stroke regardless of the stenosis level [1,2]. In contrast, stable plaques predominantly contain fibrous tissue, as well as calcifications, and are less likely to rupture and cause a cerebrovascular event [3]. Consequently, assessing plaque stability via ultrasound and biomarkers is essential for identifying patients at high stroke risk and guiding decisions on revascularization or medical therapy.

Chemokine-induced migration of inflammatory cells, such as monocytes, plays a pivotal role in both plaque formation and destabilization [4,5,6,7]. One chemokine with well-documented proatherogenic properties is MCP-1, whose levels are elevated in patients with conditions such as hypertension, obesity, and atherosclerosis [8,9]. The primary sources of MCP-1 in the vascular wall are monocytes/macrophages, fibroblasts, endothelial cells, epithelial cells, and smooth muscle cells, which may be regulated by several other cytokines and factors [10]. On the other hand, MCP-1 can be produced by adipocytes and released into circulation, especially in obesity, where large amounts of adipose tissue promote excessive secretion of mediators, including MCP-1. Obesity is also a recognized risk factor for progressive atherosclerosis and stroke [11,12,13,14]. Insulin resistance due to obesity is a risk factor for type 2 diabetes, which results in monocyte recruitment and activation, promoting further formation of atherosclerotic plaque [15]. The overproduction of cytokines significantly contributes to endothelial dysfunction—a key initiator of plaque destabilization.

Current research indicates that plasma MCP-1 levels may serve as a prognostic biomarker and an early predictor of clinical outcomes in patients with ischemic stroke [16,17]. Moreover, a genetic predisposition to elevated circulating MCP-1 has been associated with a higher risk of stroke, particularly in cases of large artery occlusion [18,19], while low plasma MCP-1 levels have been associated with a reduced risk of all-cause mortality, as well as less serious functional consequences after ischemic stroke or transient ischemic attack [20]. Elevated MCP-1 concentrations have also been positively correlated with histopathological markers of plaque instability [21].

Various imaging techniques help identify vulnerable carotid plaques, with the gray-scale median (GSM) calculated from ultrasound echogenicity being a widely used, non-invasive marker of plaque instability [22]. High GSM values correlate with stable plaques rich in fibrous tissue and calcification, whereas low GSM values indicate vulnerable plaques with high lipid content, inflammation, and hemorrhage [23]. Our recent study found significantly lower GSM values in symptomatic patients undergoing carotid endarterectomy (CEA) compared to asymptomatic individuals, and ROC analysis confirmed GSM as a useful tool for differentiating symptomatic from asymptomatic carotid disease [24].

Despite recent advances, the relationship between MCP-1 levels and plaque stability, assessed by plaque morphology using GSM analysis, remains unclear in the context of obesity. Therefore, this study aimed to evaluate the association between obesity, MCP-1 levels, and the stability of atherosclerotic plaques, as determined using the GSM scale calculated from ultrasound images, in patients undergoing CEA. Moreover, we also wanted to explore whether MCP-1 could be an independent predictor of plaque instability in this population, stratified by body mass index (BMI).

## 2. Results

Participants were stratified according to their BMI into three subgroups: normal weight (BMI < 25 kg/m^2^), overweight (BMI 25.0–29.9 kg/m^2^), and obese (BMI ≥ 30 kg/m^2^) [25]. The clinical and biochemical characteristics of the patients are presented in Table 1. Most of the analyzed parameters were comparable in the studied groups. BMI gradient was accompanied by tendency to alterations in lipid profiles. The prevalence of diabetes was higher in the obese group than in controls, and all patients with BMI ≥ 30 kg/m^2^ were hypertensive. The patients with normal weight had higher D-Dimers levels and exhibited more frequent use of aspirin compared to those with obesity. In addition, the patients with normal weight were younger than the control group.

**Figure 1:**
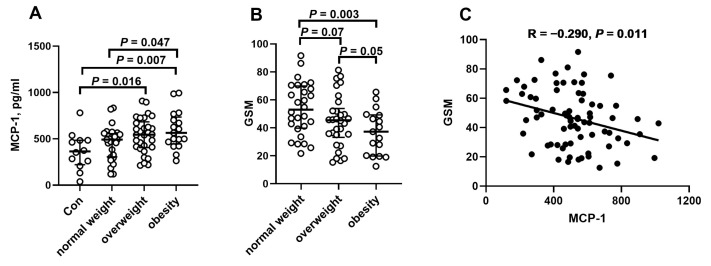
Association between MCP-1 levels, GSM score, and BMI. (**A**) MCP-1 levels in relation to BMI categories: normal weight (BMI < 25 kg/m^2^), overweight (BMI 25.0–29.9 kg/m^2^), and obesity (BMI ≥ 30 kg/m^2^). (**B**) Gray-scale median (GSM) values across BMI categories. (**C**) Correlation between MCP-1 levels and GSM score in the overall study population. Statistical significance was set at *p* < 0.05.

**Figure 2:**
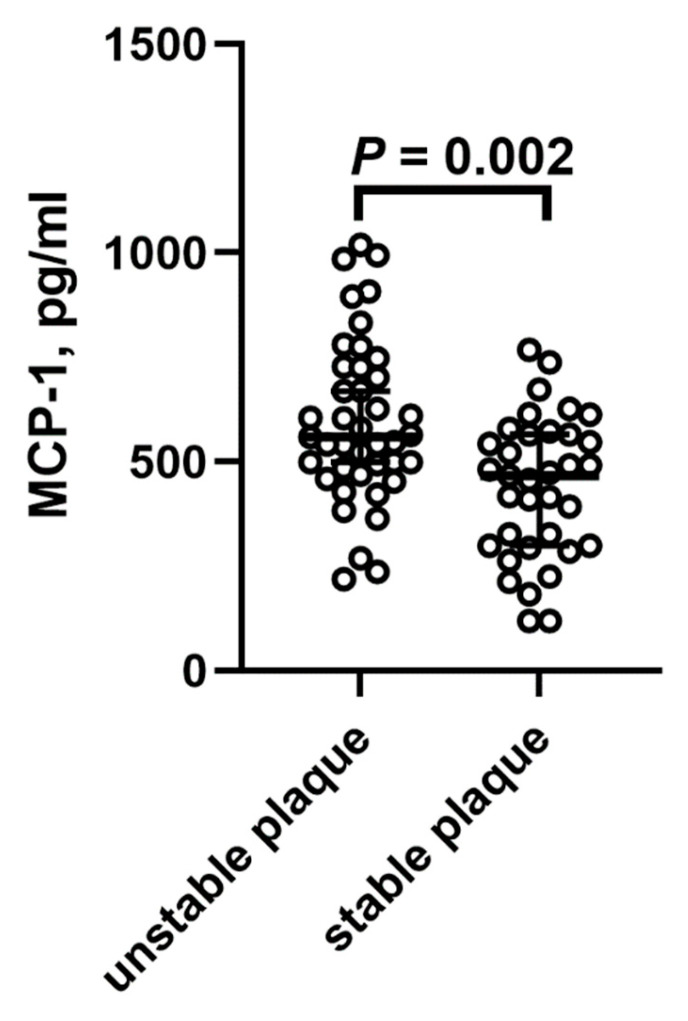
MCP-1 levels in patients with unstable and stable carotid plaques.

As shown in Figure 1A, MCP-1 levels increased with increasing body weight, whereas GSM values decreased (Figure 1B). In the overall patient group, there was an inverse correlation between MCP-1 levels and the GSM score (Figure 1C). Moreover, patients with carotid plaque instability had significantly higher MCP-1 levels than those with stable plaques (*p* = 0.002; Figure 2).

In the Spearman correlation analysis, GSM was inversely correlated with BMI (R = –0.259, *p* = 0.03) and fibrinogen levels (R = –0.236, *p* = 0.04), while it was positively associated with D-dimers (R = 0.234, *p* = 0.04) and total cholesterol levels (R = 0.276, *p* = 0.02). Patients with grade 2 hypertension exhibited lower GSM scores compared to others (40.43 ± 16.19 vs. 51.09 ± 19.67, *p* = 0.01). Additionally, patients treated with sulfonylurea derivatives showed higher GSM values than those who were untreated (59.14 ± 12.29 vs. 44.76 ± 19.04, *p* = 0.03). Conversely, patients receiving statin therapy had significantly lower GSM scores compared to those not taking this medication (43.23 ± 18.64 vs. 52.32 ± 18.24, *p* = 0.04). No significant correlations were found between the GSM score and age, sex, or other clinical and biochemical parameters in the analyzed group.

To predict plaque instability, a baseline logistic regression model was constructed using covariates that were significantly associated with the GSM score (*p* < 0.05). Logistic regression analysis identified MCP-1 as a significant factor for plaque instability in both univariate (Table 2) and multivariate models (Table 3). In univariable regression analysis, the risk factors for unstable plaque were MCP-1 (OR = 1.03, 95% CI: 1.01–1.04, *p* = 0.002), total cholesterol (OR = 0.52, 95% CI: 0.31–0.85, *p* = 0.009), the presence of grade 2 hypertension (OR = 3.52, 95% CI: 1.35–9.20, *p* = 0.01), the presence of overweight/obesity (OR = 3.44, 95% CI: 1.28–9.29, *p* = 0.01), statin use (OR = 3.45, 95% CI: 1.28–9.26, *p* = 0.01), and sulfonylurea derivative use (OR = 0.22, 95% CI: 0.04–1.13, *p* = 0.06). In multivariable regression analysis, only MCP-1 (OR = 1.04, 95% CI: 1.03–1.07, *p* = 0.009), total cholesterol (OR = 0.51, 95% CI: 0.28–0.93, *p* = 0.03), and the presence of grade 2 hypertension (OR = 3.22, 95% CI: 0.99–10.39, *p* = 0.05) were independent predictors of carotid plaque instability (Table 3).

## 3. Discussion

This study is the first to examine the relationships between obesity, serum MCP-1 levels, and carotid plaque characteristics assessed using the ultrasonographic GSM scale, as well as the impact of this interplay on plaque stability in patients undergoing carotid endarterectomy (CEA). The findings indicate that the obesity-related MCP-1 serves as an independent predictor for carotid plaque instability.

In the present study, MCP-1 levels increased with body mass, whereas GSM decreased significantly as obesity developed, and we demonstrated a clear inverse correlation between MCP-1 levels and the GSM score. Moreover, the patients with unstable plaques had significantly higher MCP-1 levels compared to those with stable plaques. These results indicate that obesity, by regulating the level of MCP-1, may adversely affect the stability of atherosclerotic plaque. MCP-1 plays a crucial role in atherosclerosis by stimulating monocyte infiltration into the subendothelial space, where they transform into foam cells, subsequently forming fatty streaks and, ultimately, atherosclerotic plaques. These proatherogenic properties of MCP-1 are well documented [8,9,10], and the elevated circulating level of this chemokine was associated with a higher risk of stroke [18] and with histopathological markers of plaque instability [21]. Moreover, MCP-1 levels were increased in the blood of patients with ischemic stroke a few hours after the stroke symptoms occurred, and it was independently related to clinical outcome scores at specific time points [17]. Obesity is also a well-established risk factor for stroke, independently from the presence of other known cardiovascular risk factors [11,12,13]. However, some studies reported significantly lower mortality rates in stroke patients with higher BMI values [26,27,28].

In this study, we found that MCP-1 was an independent predictor of plaque instability in our univariate and multivariate logistic regression analyses; however, BMI itself was not. Although the presence of overweight/obesity independently served as a predictor of plaque instability in univariate logistic regression analysis, it was unable to predict this in the multivariate model. These data indicate that not obesity itself, but an obesity-induced increase in MCP-1 concentrations was enough to independently predict carotid plaque instability. In obese patients, excessive MCP-1 production by adipocytes may promote atherosclerotic plaque formation by exacerbating inflammation and monocyte migration [29]. Furthermore, MCP-1 exhibits angiogenic effects on endothelial cells [30], potentially contributing to adipose tissue expansion and remodeling. A recent study demonstrated that MCP-1 induces endothelial cell apoptosis [31], which may explain both the accelerated atherosclerotic process and the development of a more necrotic, apoptosis-rich plaque core in these patients, ultimately leading to increased plaque instability. The prognostic value of MCP-1 measured in plasma versus within atherosclerotic plaques concerning plaque instability has been postulated by some authors. In the large-scale study, Georgakis et al. [21] found that the higher levels of MCP-1 in atherosclerotic plaques were independently associated with histological features of plaque instability, including a large lipid core, high macrophage content, low collagen and smooth muscle cell content, and the presence of intraplaque hemorrhage. Furthermore, patients with higher plaque MCP-1 levels experienced a higher risk of major adverse cardiovascular events within 30 days post-procedure. However, there was no significant correlation between plasma and intraplaque MCP-1 levels [21]. De Lemos et al. [32] showed that elevated circulating MCP-1 levels are associated with worse long-term outcomes in patients with acute coronary syndrome, including increased risk of death or myocardial infarction at 10 months of follow-up. However, the intraplaque MCP-1 levels were not measured in this study. The recent research of Yuan et al. [33] found that patients with vulnerable plaque had higher serum concentrations of CCL2/MCP-1 compared to those with stable plaque, identifying this chemokine as a risk factor for plaque stability.

We identified reduced total cholesterol level as another independent predictor of plaque instability in the studied patients. Although elevated cholesterol has long been associated with a risk factor for coronary heart disease [34], its role in stroke remains controversial. Although the associations between high serum total cholesterol levels and an increased risk of ischemic stroke have been reported [35,36], observational studies have not shown a clear relationship between cholesterol levels and stroke [37,38]. Another cohort study even indicated that low cholesterol levels significantly increased the risk of stroke [39]. Moreover, recent studies have demonstrated that low values of total cholesterol are associated with poor outcomes in patients with acute ischemic stroke and with poor overall health status [40,41,42], especially in older people—characterizing a phenomenon called “the cholesterol paradox” [43]. Herein, we noticed a tendency for total cholesterol to decrease as BMI increased (Table 1), whereas the statin treatment was a risk factor for plaque instability in univariate logistic regression analysis (Table 2). The majority of the patients in each studied group were treated with statins, so the reduced levels of total cholesterol can reflect the effect of these lipid-lowering drugs on plaque stability. Statin therapy can reduce plaque vulnerability not just by lowering lipid levels but also through other pleiotropic effects [44]. Simultaneously, some vascular responses to statin therapy may be unfavorable, especially in older people [45]. Consistent with the findings of these previous studies, we recently showed that total cholesterol positively correlated with GSM score in patients undergoing CEA, whereas statin therapy was associated with an increased level of endothelin-1, which turned out to be a factor related to carotid plaque instability [46]. It has been demonstrated that smoking, along with the resulting enhanced inflammatory response, is one of the major risk factors for the formation of atherosclerotic plaques [47]. This is directly reflected in the profile of our hospitalized patients, the majority of whom are current or former smokers. Additionally, the vast majority of participants in our study are over 60 years of age, and when they were young, smoking was a widespread trend. Many of them remained smokers, which is reflected in our study population.

The results of both univariate and multivariable logistic regression analysis found that grade 2 hypertension was independently related to carotid plaque instability. Hypertension was present in the majority of our patients, and grade 2 hypertension was the most prevalent in each studied group. Nowadays, it is known that hypertension is one of the important risk factors for ischemic stroke [48,49]. Fassaert et al. [50] found that preoperative hypertension was associated with the presence of macrophages, a lipid core, and intraplaque hemorrhage—the markers of unstable carotid plaque in severely atherosclerotic patients undergoing CEA. Other studies have shown that hypertension was associated with both plaque volume and progression [51,52]. A recent study evaluated the predictive value of MCP-1 in individuals with hypertension, with and without stroke. The findings indicated that higher MCP-1 levels could serve as a strong predictor of stroke in hypertensive individuals [53], and our results are in line with these findings.

### Limitations

The limitations of this study include its cross-sectional design and the relatively small number of patients in the subgroups, as well as the numerical predominance of men over women. However, the predominance of men in our study may seemingly be only a limiting factor. In the Caucasian population, the prevalence of carotid atherosclerotic disease—defined as ≥50% stenosis of the carotid arteries—increases with age and is higher in men [54]. This is supported by a recent meta-analysis that included 99,495 patients with carotid artery disease (35,160 women and 64,335 men) [54]. The main limitation of ultrasound techniques is their operator-dependent nature, which can reduce their accuracy, introduce difficulties in the manual delineation of hypoechoic plaques, and affect inter-observer agreement. To diminish the effect of these factors, all examinations were performed by an experienced ultrasonographer (A.P.), following the protocol of standard clinical settings. Moreover, as patients undergoing carotid artery stenting were not included in this study, results cannot be extrapolated for such patients. Despite these limitations, the conclusions of our study may have clinical implications. Patients were assigned to the stable or unstable carotid plaque group based on the previously designated optimal cut-off point of GSM [24], allowing for a more accurate distinction between patients with stable and unstable plaques. All patients included in the study had complete information regarding laboratory data, comorbidities, and medications taken. However, multicenter studies or larger cohorts are needed to assess the clinical implications of our data.

## 4. Materials and Methods

### 4.1. Patients

A total of 77 patients who underwent carotid endarterectomy (CEA) in the Department of Vascular Surgery and Transplantation at the Medical University of Bialystok were included in this study. In the study group, atherosclerotic plaques caused internal carotid artery stenosis ranging from 50% to 99%, as assessed by angio-CT based on the NASCET criteria [55]. The general inclusion criteria for carotid endarterectomy (CEA) were symptomatic patients with ≥50% carotid stenosis and asymptomatic patients with ≥70% carotid stenosis, applicable to both women and men, in accordance with the European Stroke Organization guidelines [56]. Exclusion criteria included cerebral hemorrhage, intracranial tumors, intracranial aneurysms, heart failure, atrial fibrillation, and severe liver dysfunction. Based on an optimal cut-off for the GSM score [24], unstable carotid plaques were identified in 40 individuals (52%), while stable plaques were observed in 37 participants (48%). The control group consisted of 12 non-obese patients who qualified for endovascular aneurysm repair (EVAR) of abdominal aortic aneurysms. All control subjects underwent preoperative angio-CT scan, as well as Doppler ultrasound examination of the aorta, carotid, iliac, and vertebral arteries, and only patients without atherosclerotic changes in the examinations mentioned above were included in the study.

The study was approved by the Ethics Committee of the Medical University of Bialystok (APK.002.390.2020), and written informed consent was obtained from all participants. Definitions of comorbidities and a detailed description of the GSM measurement have been reported previously [24].

### 4.2. Biochemical Assessment

Biochemical analyses were performed using blood samples collected one day before the CEA procedure. All blood samples were drawn in the morning after an overnight fast. All individuals who participated in the present study had their blood samples collected for biochemical determination under the same standardized conditions—between 8:00 and 9:00 a.m., but before breakfast. This procedure eliminated the influence of feeding status on MCP-1 results. Serum MCP-1 levels were quantitatively determined using the enzyme-linked immunosorbent assay technique (ELISA; Human CCL2/MCP-1 Quantikine ELISA, R&D Systems, a Bio-Techne brand, Minneapolis, MN, USA). This assay is designed to measure MCP-1 in various biological materials, including cell culture supernatants, serum, plasma, and urine. The intra- and inter-assay coefficients of variation were 4.7% and 5.8%, respectively. Serum concentrations of glucose, D-dimer, total cholesterol, HDL-cholesterol, and triglycerides were measured using a Cobas C311 biochemical analyzer (Roche, Mannheim, Germany). Serum LDL-cholesterol concentrations were calculated using Friedewald’s formula. Serum C-reactive protein (CRP) levels were determined using a nephelometric technique (Beckman Coulter Image 800; Fullerton, CA, USA).

### 4.3. Statistical Analysis

The normality of the distribution was assessed using the Shapiro–Wilk test. Categorical variables are expressed as percentages (%), and continuous variables are presented as means (standard deviations) or medians with interquartile ranges (IQRs), depending on their distribution. The Chi-squared test was used to compare qualitative data. For normally distributed continuous variables, one-way analysis of variance (ANOVA) with Tukey’s post hoc test was employed, while the Kruskal–Wallis test with Dunn’s post hoc test was used for non-normally distributed variables. Spearman correlation analysis was performed to evaluate the relationship between the GSM score and clinical and laboratory parameters. Stepwise logistic regression analysis was conducted to estimate the association between plaque instability (unstable vs. stable), MCP-1, and other clinical and biochemical variables that were significantly correlated with the GSM score in univariate analysis. Odds ratios (ORs) are presented with 95% confidence intervals (CIs). A two-sided *p*-value of less than 0.05 was considered statistically significant. Statistical analyses were performed using Statistica version 13.1 (StatSoft, Tulsa, OK, USA) and GraphPad Prism version 10.2.2 (GraphPad Software, La Jolla, CA, USA).

## 5. Conclusions

This is the first observation linking obesity-related MCP-1 with carotid plaque instability in patients undergoing CEA. Elevated MCP-1 levels independently predict carotid plaque instability, particularly in overweight and obese patients with hypertension, suggesting its potential as a biomarker for assessing the risk of vascular events in this population. The coexistence of both atherosclerosis and hypertension in obese patients is not uncommon. Effective antihypertensive therapy can significantly reduce cardiovascular risk in these patients by mitigating endothelial damage caused by blood flow; however, its protective effect against inflammatory chemokines appears to be insufficient [57]. These findings highlight the urgent need for novel therapies targeting inflammatory chemokines to reduce vascular risk, even in patients receiving effective antihypertensive treatment. Therapeutic strategies targeting MCP-1 may offer a promising approach for treating patients with carotid artery disease. However, high-quality clinical studies involving large patient cohorts are necessary to confirm their efficacy and safety. To date, therapies aimed at blocking the MCP-1 receptor, as well as chemokine-neutralizing molecules (neutraligands) targeting MCP-1, have shown encouraging results [58]. MCP-1 has emerged as a promising therapeutic target for the treatment and prevention of immune-mediated diseases, cancer, and neuropathic pain [58]. Integrating MCP-1 assessment into routine diagnostics could sharpen the identification of high-risk patients and aid in optimizing therapeutic strategies, especially in those with obesity and hypertension. It is important to emphasize that patients—particularly those with 50–69% carotid artery stenosis—require a personalized approach, as the decision regarding surgical intervention is often complex. A subgroup that warrants special attention includes asymptomatic or minimally symptomatic patients within this stenosis range. In many clinical settings, the decision to proceed with revascularization in such cases is frequently subjective, based primarily on the degree of stenosis, without sufficient consideration of objective predictors or plaque instability. While intervention is generally recommended for symptomatic patients, the decision to operate on asymptomatic individuals is more nuanced due to the associated risk of surgical complications. However, postponing surgery for patients who exhibit morphological features of plaque instability—especially when accompanied by elevated MCP-1 levels—may significantly increase the risk of future stroke. Therefore, timely surgical intervention, ideally before a cerebrovascular event occurs, is crucial. Furthermore, optimized medical therapy has been shown to substantially reduce stroke risk in patients with asymptomatic internal carotid artery (ICA) stenosis. The combined use of our plaque stability assessment tool—the gray-scale median (GSM)—alongside MCP-1 level evaluation in asymptomatic or minimally symptomatic patients may significantly enhance the decision-making process for surgical revascularization, particularly in cases with borderline stenosis severity.

Our results suggest that targeting MCP-1-related pathways could offer novel therapeutic strategies to prevent plaque rupture and ischemic events in patients with severe carotid atherosclerosis. Further studies with larger cohorts and mechanistic investigations are needed to validate these findings, explore the clinical applicability of MCP-1 measurement in risk stratification, and optimize treatment in this population.

## Figures and Tables

**Table 1:** Clinical and biochemical characteristics of participants.

	Controls n = 12	Normal Weight n = 28	Overweight n = 32	Obesity n = 17
Age, years	73.5 (8.39)	66.46 (7.55) ^a^	69.48 (10.09)	67.41 (7.09)
Male sex, %	83.3	60.7	83.9	88.2
BMI, kg/m^2^	24.43 (1.43)	22.52 (1.85)	27.51 (1.41) ^a,d^	32.68 (2.16) ^b,d,e^
Fibrinogen, g/L	3.45 (2.93–4.18)	3.91 (3.21–4.66)	3.76 (3.42–4.09)	3.95 (3.50–4.69)
D-dimers, μg/mL	0.40 (0.19–0.63)	0.49 (0.35–0.71)	0.38 (0.27–0.72)	0.35 (0.27–0.62) ^c^
TC, mmol/L	4.44 (3.57–4.84)	4.19 (3.54–5.35)	3.88 (3.42–4.94)	3.74 (2.91–4.58)
LDL, mmol/L	2.59 (1.73–2.82)	2.35 (1.93–3.49)	2.15 (1.79–3.21)	1.99 (1.65–2.56)
HDL, mmol/L	1.31 (0.75–1.45)	1.19 (0.96–1.58)	1.06 (0.98–1.27)	0.92 (0.80–1.24)
TG, mmol/L	1.08 (0.91–2.15)	1.37 (0.93–1.66)	1.25 (1.05–1.57)	1.52 (1.26–1.87)
CRP, mg/L	3.40 (1.50–8.10)	2.70 (0.80–10.20)	2.45 (1.20–4.60)	3.40 (2.40–6.65)
Glucose, mmol/L	4.22 (3.64–5.11)	4.78 (4.03–5.58)	5.06 (4.06–6.06)	5.00 (4.67–5.94)
Hypertension, %	75.0	85.7	81.3	100
Grade 1 hypertension, %	16.7	35.7	21.9	47.1
Grade 2 hypertension, %	58.3	35.7	46.9	47.0
Grade 3 hypertension, %	0	14.3	12.5	5.9
CKD, %	16.7	10.7	19.4	11.8
IHD, %	25.0	21.5	25.8	17.6
DM, %	16.7	25.0	32.3	41.2 ^a^
Smoking, %	75.0	71.4	80.6	82.4
Diuretics, %	33.3	21.4	41.9	29.4
RAAS inhibitor, %	83.3	67.9	67.7	88.2
β-blockers, %	58.3	39.3	48.4	47.1
CCA, %	16.7	39.3	38.7	23.5
Aspirin, %	83.3	85.7	64.5	52.9 ^c^
Statins, %	41.7	67.9	61.3	64.7

Categorical data are presented as percentage (%), continuous variables are presented as mean (SD) if distributed normally or as median (IQR) for non-normally distributed data; ^a^ *p*< 0.05, ^b^ *p* < 0.001 controls vs. others; ^c^ *p* < 0.05, ^d^ *p* < 0.001 normal weight vs. others; ^e^ *p* < 0.001 overweight vs. obesity. Abbreviations: BMI, body mass index; TC, total cholesterol; LDL, low-density lipoprotein cholesterol; HDL, high-density lipoprotein cholesterol; TG, triglycerides; CRP, C-reactive protein; CKD, chronic kidney disease; IHD, ischemic disease; DM, diabetes; RAAS, renin–angiotensin–aldosterone system; and CCA, calcium channel antagonist.

**Table 2:** Univariate logistic regression analysis for assessment of predictors of carotid plaque instability.

	OR	(95% CI)	*p*
MCP-1	1.03	1.01–1.04	0.002
Total cholesterol	0.52	0.31–0.85	0.009
Grade 2 hypertension	3.52	1.35–9.20	0.010
Overweight + obesity	3.44	1.28–9.26	0.014
Statin	3.45	1.25–9.36	0.015
Sulfonylurea derivatives	0.22	0.04–1.13	0.063
BMI	1.09	0.97–1.22	0.149
Fibrynogen	1.003	0.99–1.01	0.304
D-dimers	0.74	0.33–1.65	0.742

MCP-1, monocyte chemoattractant protein-1; BMI, body mass index.

**Table 3:** Multivariate logistic regression analysis for assessment of predictors of carotid plaque instability.

	OR	(95% CI)	*p*
MCP-1	1.04	1.03–1.07	0.009
Total cholesterol	0.51	0.28–0.93	0.028
Grade 2 hypertension	3.22	0.99–10.39	0.050

MCP-1, monocyte chemoattractant protein-1.

## Data Availability

Data is contained within the article.

## Abbreviations

The following abbreviations are used in this manuscript:

BMI	Body mass index
CEA	Carotid endarterectomy
ELISA	Enzyme-linked immunosorbent assay
EVAR	Endovascular aneurysm repair
GSM	Gray-scale median
MCP-1	Monocyte Chemoattractant Protein-1
TC	Total cholesterol
HDL	High-density lipoprotein cholesterol
LDL	Low-density lipoprotein cholesterol
TG	Triglycerides
RAAS	Renin–angiotensin–aldosterone system
CRP	C-reactive protein
CKD	Chronic kidney disease
IHD	Ischemic disease
DM	Diabetes
CCA	Calcium channel antagonist
